# Valproic Acid Application to Modify Post Surgical Fibrosis in a Model of Minimally Invasive Bleb Surgery

**DOI:** 10.1167/tvst.14.6.6

**Published:** 2025-06-02

**Authors:** Phey Feng Lo, Merry Gunawan, Zhu Li Yap, Carol Leong, Fuxuan Kwek, Jayasudha Varadarajan, Xiaomeng Wang, Masaaki Kageyama, Tina T. Wong

**Affiliations:** 1 Singapore Eye Research Institute, Singapore, Singapore; 2 Santen-SERI Open Innovation Centre, Singapore, Singapore; 3 Singapore National Eye Centre, Singapore, Singapore; 4 Institute of Molecular and Cell Biology, A*STAR, Singapore, Singapore; 5 Duke-NUS Medical School, Singapore, Singapore

**Keywords:** valproic acid, minimally invasive bleb surgery, surgical fibrosis

## Abstract

**Purpose:**

To evaluate different routes of valproic acid (VPA) administration in modulating various phases of wound healing events after minimally invasive bleb surgery (MIBS).

**Methods:**

Thirty New Zealand White rabbits underwent surgical implantation of PRESERFLO MicroShunts. Animals were divided into treatment groups of mitomycin C (MMC) 0.4 mg/mL, MMC 0.1 mg/mL, VPA 1 mg/mL, and VPA 30 mg/mL. Treatments with topical and subconjunctival administration were investigated in the combination groups of low-dose MMC (0.1 mg/mL) and VPA. IOP was measured and slit-lamp photographs were taken weekly. Fibrotic gene expression was analyzed in the bleb by quantitative polymerase chain reaction and western blot. Histology was performed following sacrifice on day 28.

**Results:**

Low-dose MMC (0.1 mg/mL) combined with VPA resulted in prolonged bleb survival to day 28. This was associated with reduced collagen 1 and fibronectin gene and protein expression (*P* < 0.05). There was also a favorable extracellular matrix structure on histological analysis due to the presence of fine immature collagen fibrils surrounding the shunt opening. Intraocular pressure lowering was seen, with a reduction of 20% from baseline IOP until day 14. Both topical and subconjunctival administration of VPA demonstrated similar outcomes, with no overt side effects observed throughout the experimental period at different doses or dosing regimens.

**Conclusions:**

VPA is a versatile and effective adjunctive agent that can be used in combination with low-dose MMC to produce favorable bleb characteristics in MIBS.

**Translational Relevance:**

VPA is both efficacious and safe as an antifibrotic agent, and different routes of administration are available to target different stages of fibrosis after filtration surgery.

## Introduction

Surgical management of glaucoma is very effective in lowering intraocular pressure (IOP) to further reduce the risk of disease progression and optic nerve damage. With the introduction of minimally invasive glaucoma surgery (MIGS), there is now a shift toward early intervention for mild to moderate disease. MIGS provides a good safety profile and rapid recovery that are associated with efficacious IOP lowering, which reduces the drop burden in the early stages of the disease. Traditional glaucoma filtration surgery and glaucoma drainage devices are the surgical procedures of choice for more moderate to advanced disease, where a much lower target IOP, often in the low teens to single digits, is desirable. Subconjunctival MIGS, often referred to as minimally invasive bleb surgery (MIBS), has been reported to achieve comparable IOP reduction compared to traditional filtration surgeries while maintaining a good safety profile.^1^

The PRESERFLO MicroShunt (Santen Pharmaceutical, Osaka, Japan) allows aqueous humor to drain from the anterior chamber to the subconjunctival space.^2^ In glaucoma filtration surgery, IOP control is achieved with the adjunctive use of mitomycin C (MMC) to reduce subconjunctival fibrosis from Tenon's fibroblast activity and proliferation.^3^ However, MMC is cytotoxic and causes non-specific and extensive cell death, leading to complications such as bleb leaks, hypotony, and endophthalmitis.^4^^,^^5^ It has also been reported that, despite MMC treatment, fibroblasts can retain features of wound-healing behavior.^6^

Several agents have been explored and reported as adjunctive antifibrotics in glaucoma filtration surgery, although none has been widely accepted.^7^^,^^8^ Valproic acid (VPA) is a small molecule drug widely used in neurological diseases with additional repurposed anti-fibrotic properties identified.^9^ We have previously shown that subconjunctival injection of VPA was effective in reducing collagen production, promoting good bleb function in a rabbit model of MIBS.^10^ Furthermore, subconjunctival application of VPA used as an adjunct with low-dose MMC was found to be a safer and more efficacious option in maintaining bleb vascularity and lowering IOP compared to MMC or VPA treatment alone.^11^

 In this current study, we explored different routes of VPA administration both subconjunctivally and topically to determine the most optimal mode of delivery in modulating wound healing events after experimental MIBS.

## Materials and Methods

### Rabbit Model of MIBS

New Zealand White (NZW) rabbits weighing 2.0 to 2.4 kg, 12 to 14 weeks old, were acclimatized for 20 days before surgery at the SingHealth Experimental Medical Centre (Singapore General Hospital). All animal experiments were approved by the Institutional Animal Care and Use Committee (2022/SHS/1735) and treated in accordance with the ARVO Statement for the Use of Animals in Ophthalmic and Vision Research.

The surgical procedure involving the PRESERFLO MicroShunt was performed in rabbits as previously described.^10^ The rabbits were anesthetized with a combination of ketamine (Ketaset; Fort Dodge Animal Health, Southampton, UK) and medetomidine HCl (Domitor; Pfizer Animal Health, Sandwich, UK). A fornix-based conjunctival flap was raised, and blunt dissection of the subconjunctival space was performed approximately 3 mm along the limbus and 5 mm posteriorly. Then, 0.2 mL of basal salt solution on a 30-gauge needle was injected to deepen the anterior chamber (AC) and the PRESERFLO MicroShunt was inserted per the manufacturer's instructions. The PRESERFLO MicroShunt is an implantable glaucoma drainage device made from a flexible poly(styrene-*block*-isobutylene-*block*-styrene) polymer with an outer diameter tube of 350 µm and a lumen of 70 µm. Triangular fins on the implant prevent migration of the tube into the AC. The device is designed for implantation under the subconjunctival/Tenon’s space. A 1-mm blade is used to create the start of a sclerostomy 2.5 mm behind the limbus, followed by tunnel entry with a 25-gauge needle into the AC to complete the sclerostomy. The implant then sits 2.5 mm in the AC and 3 mm on the sclera within the conjunctival pocket. Under experimental conditions involving MMC treatment, MMC has been applied with a surgical sponge soaked in specified doses of MMC solution. The sponge was placed within the conjunctival/Tenon's flap for 2 minutes before removal, then carefully and rigorously rinsed with 10 to 20 mL of saline solution, followed by insertion of the MicroShunt as per the manufacturer's instructions. Watertight closure of the conjunctival incision was performed with purse-string sutures using 10–0 nylon and, where required, a mattress suture. Only the left eye of each rabbit was operated on, and the surgical procedure was performed superiorly in each animal. One drop each of guttae chloramphenicol and Betnesol-N (Glaxo Wellcome, Uxbridge, UK) ointment was instilled at the end of surgery.

**Figure 1.**
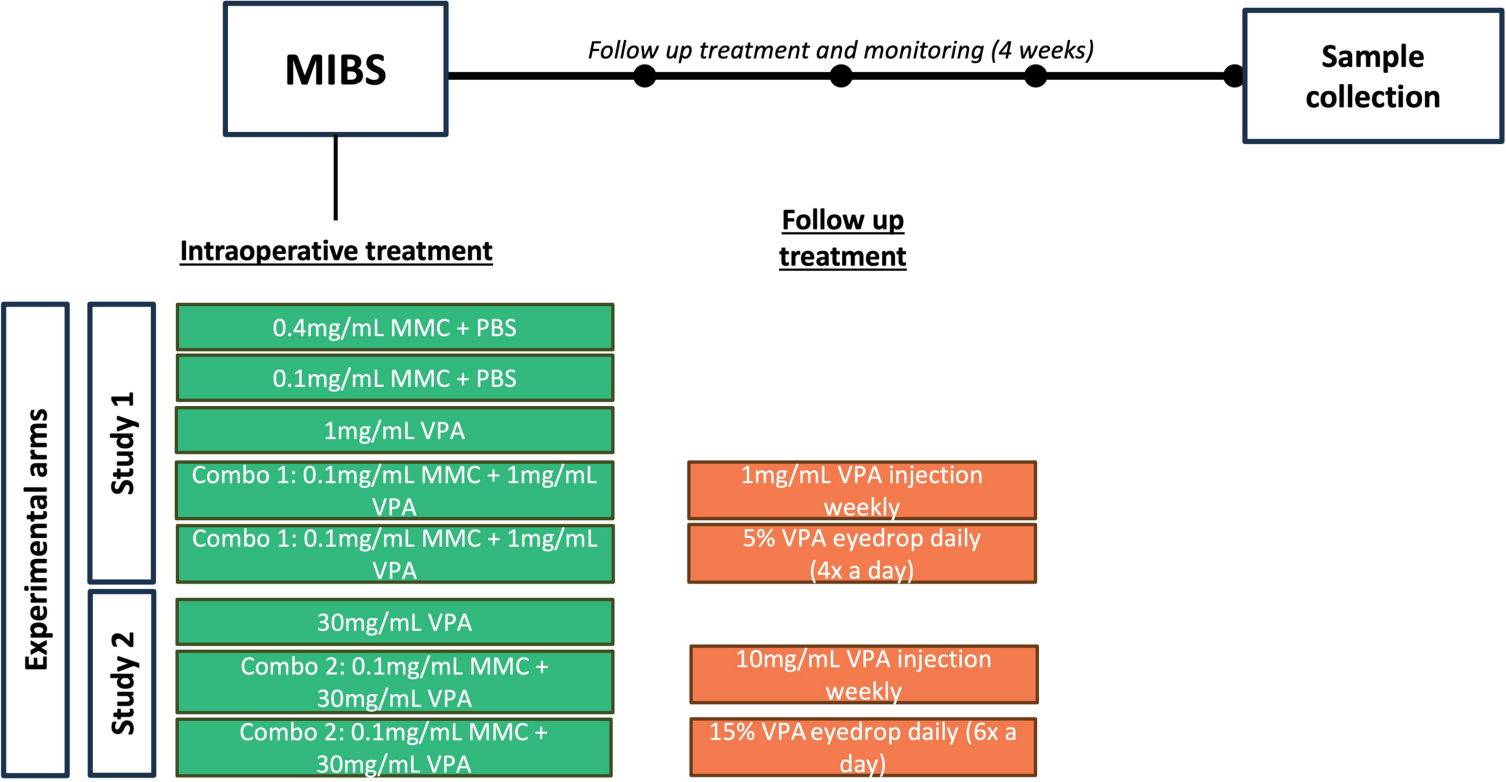
Experimental arms.

Treatment conditions tested were as shown in Figure 1 (Study 1), as follows: group 1, 0.4-mg/mL MMC; group 2, 0.1-mg/mL MMC; and group 3, VPA (operated eyes were treated with 1× subconjunctival (SC) injection of 0.1 mL of 1-mg/mL VPA at the following postoperative time points: days 0, 1, 2, 3, 4, 5, 6, 7, 10, 14, and 21). Two initial combination groups consisted of the operated eye receiving 0.1-mg/mL MMC with SC injections of 0.1 mL of 1-mg/mL VPA at day 0, followed by SC injection of 0.1 mg of 1-mg/mL of VPA at days 7, 14, and 21 (group 4) or 5% w/v VPA eye drops applied four times daily from day 1 to day 28 (group 5). Five NZW rabbits were used for all treatment conditions described; however, one NZW rabbit from group 1 was excluded due to surgical complications.

In addition, a second experiment (as shown in Fig. 1, Study 2) evaluated a formulation containing high-dose VPA (30 mg/mL) with 1× SC injection at 0.1 mL, given intraoperatively without further treatment as well as two further combination groups. In the combination groups, eyes received either MMC (0.1 mg/mL) plus SC injection of VPA (30 mg/mL) at the time of surgery, followed by SC injection of 0.1 mL of VPA (10 mg/mL) at days 7, 14, and 21, or topical eye drops (15% w/v) six times daily from day 1 to day 28 (Fig. 1). Six NZW rabbits were used for all treatment conditions described; however, one rabbit from the 0.1-mg/mL MMC treatment group was excluded due to infection.

### IOP Measurement on Rabbits

IOP was measured with a pneumatonometer (Reichert Technologies, Buffalo, NY) after the application of topical anesthesia with 0.5% proparacaine hydrochloride (Alcon, Geneva, Switzerland). The pneumatonometer was calibrated for the eye of the rabbit. The animals were acclimatized to the IOP measurements for 2 weeks before actual experiments. Post-surgery IOP was measured twice weekly up to day 27. Measurements were performed at the same time each day, and three readings were taken at each time point. Rabbits were conscious throughout the IOP measurements.

### Primary Human Tenon's Capsule Fibroblast Isolation and Culture

Primary human Tenon's fibroblasts (HTFs) were isolated from subconjunctival Tenon's capsule tissue excised during glaucoma filtration surgery, as described previously.^12^ Patients were consented for tissue donation to research prior to glaucoma filtration surgery. Written informed consent was provided by all patients.

Explanted tissue was carefully attached to the bottom of a six-well plate (Greiner Bio-One, Jena, Germany) and HyClone Dulbecco's Modified Eagle's Medium (DMEM, SH30243-01; Cytiva, Marlborough, MA) with high glucose, supplemented with Gibco 10% fetal bovine serum (FBS, 10082147; Thermo Fisher Scientific, Waltham, MA) and 1% penicillin/streptomycin (09367-34, Nacalai Tesque, Kyoto, Japan). When the HTF monolayers reached confluence, cells were passaged and subcultured for subsequent experiments. Cells were maintained in a humidified incubator at 37°C with 5% CO_2_.

### Real-Time Quantitative Polymerase Chain Reaction

Conjunctival tissues were processed and analyzed as described previously. Briefly, for mRNA analyses of the rabbit model, a small portion of the operated conjunctiva was excised on the day of sacrifice (day 28). A similar area of unoperated conjunctiva in the contralateral eye of each rabbit was harvested to obtain a baseline value for the calculation of fold change in transcript expression. Both rabbit conjunctival tissues and fibroblasts were processed and analyzed by quantitative polymerase chain reaction (qPCR) as described previously.^10^ Samples were collected in RNAlater solution (Life Technologies, Carlsbad, CA) and lysed using the Vibra-Cell VCX 130 ultrasonic processor (Sonics & Materials, Newtown, CT). Total RNA was recovered with Invitrogen TRIzol Reagent (Thermo Fisher Scientific) and the RNeasy kit (QIAGEN, Hilden, Germany) as described previously. Total RNA was reverse transcribed into cDNA using random Invitrogen hexamer primers with Invitrogen SuperScript III Reverse Transcriptase (Thermo Fisher Scientific). All qPCR reactions utilized Power SYBR Green PCR Master Mix (Applied Biosystems, Foster City, CA), were performed in triplicate in volumes of 10 µL in 384-well microtiter plates, and were run using the LightCycler 480 System (Roche Diagnostics, Indianapolis, IN). All mRNA levels were measured as cycle threshold (CT) levels. *Rna18s1* was determined to be the most suitable housekeeping gene of the four analyzed (*Actb*, *Rna18s1*, *Gapdh*, and *Rpl13a*) using NormFinder software.^10^ The value for each operated eye was calculated as fold change relative to the corresponding contralateral unoperated eye by the 2^−∆∆CT^ method. Primers used for rabbit tissue samples were *Rna18s1*: forward, 5′-CTTTGGTCGCTCGCTCCTCTCC-3′, and reverse, 5′-TCTGATAAATGCACGCATCCCACAC-3′; Col1a1: forward, 5′-CGATGGCTTCCAGTTCGAGT-3′, and reverse, 5′-CTACGCTGTTCTTGCAGTG-3′; Fn1: forward, 5′-GGATGTTCCCTCCACAGTTCA-3′, and reverse, 5′-TGGTCCGCCTAAAACCATGT-3′.

### Immunoblotting

Conjunctival tissue lysates were resolved by sodium dodecyl sulfate–polyacrylamide gel electrophoresis (SDS-PAGE) followed by immunoblotting as previously described.^13^ Anti-type I collagen was obtained from MD Bioproducts (St. Paul, MN). The antibody for glyceraldehyde 3-phosphate dehydrogenase (GAPDH) was obtained from Santa Cruz Biotechnology (Dallas, TX). Horseradish peroxidase–conjugated secondary antibodies were obtained from Jackson ImmunoResearch (West Grove, PA). Densitometric analyses, where potential errors in loading were corrected to levels of the housekeeping GAPDH, were performed as reported previously.^13^

### TUNEL Assay

Conjunctival cryosections from the bleb area were examined for the presence of degraded DNA using the Invitrogen Click-iT Plus TUNEL Assay Kit. Apoptotic cells were identified by staining with a secondary antibody conjugated to Alexa Fluor 594 Picolyl Azide dye. Nuclei were visualized by staining with Hoechst 33342. Labeled tissues were observed under an Axio Imager M2 microscope (Carl Zeiss Microscopy, Oberkochen, Germany).

### Cell Viability Assay

**Figure 2.**
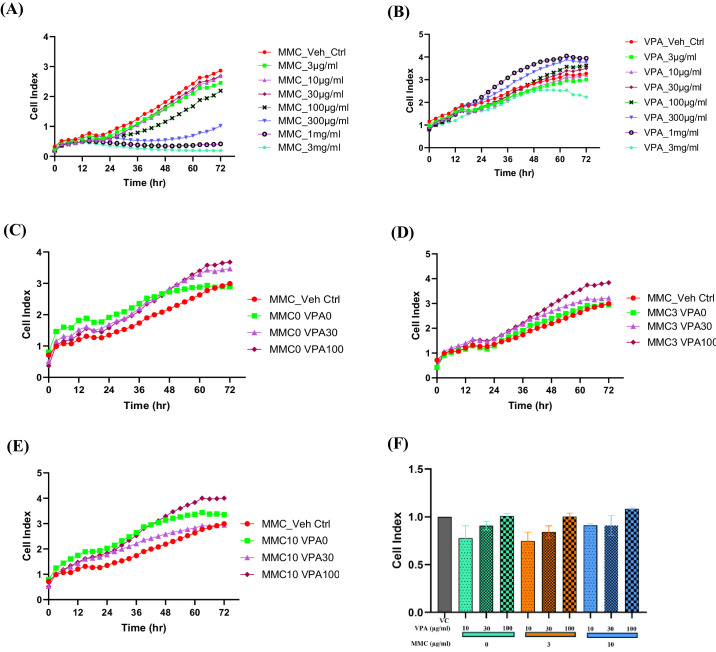
xCELLigence real-time cell analysis of primary HTFs treated with increasing doses of MMC or VPA alone (**A**, **B**) or their combination (**C**–**F**). (**A**) HTFs were treated with different concentrations of MMC (3–3000µg/mL) for 4 minutes, followed by phosphate-buffered saline (PBS) washes twice and incubated in fresh cell culture media for 72 hours. (**B**) HTFs were treated with different concentrations of VPA (3–3000µg/mL) for 72 hours. (**C**–**E**) Cells were treated with different concentrations of MMC (0, 3, and 10 µg/mL) for 4 minutes, followed by PBS washes twice and subsequent treatment with different concentrations of VPA (0, 30, and 100 µg/mL) for 72 hours. (**F**) Cell viability at 72 hours following the treatment with different MMC and VPA combinations, normalized to the vehicle control. Data are presented as mean ± SD for three independent experiments. Statistical significance was determined using one-way ANOVA followed by Dunnett's multiple-comparisons test, and all the treatment groups were non-significant.

The effect of drug treatment on HTF viability was evaluated using the xCELLigence MP real-time cell analyzer (Agilent, Santa Clara, CA) following the manufacturer's instructions. HTFs were seeded in triplicate onto E-Plate 96-well plates (Roche Diagnostics) at a density of 5000 cells per well, with vehicle control (Gibco Dulbecco's Phosphate-Buffered Saline; 14190094, Thermo Fisher Scientific) or varying doses of MMC, VPA, or a combination of MMC and VPA, as specified in Figure 2.

Cell-sensor impedance was continuously monitored over 72 hours, and data were expressed as the cell index, an arbitrary unit reflecting changes in cell viability.

### Immunohistochemical Evaluation of Cryosections

During enucleation, the upper lid was removed with the whole eye to preserve the bleb and superior conjunctiva. Each tissue sample was fixed with paraformaldehyde and then placed in a slurry of optimal cutting temperature compound in cryomold before freezing in dry ice and storage in a −80°C freezer until ready for sectioning using the Microm HM550 (Carl Zeiss Microscopy). Cryosections (5 µm) were then subjected to histochemical or immunofluorescent analyses. Histochemical evaluation of operated conjunctival cryosections of the bleb area was performed by staining with hematoxylin and eosin (H&E) or Picrosirius red, as described previously.

Polarization microscopy for Picrosirius red–stained cryosections was performed using the Eclipse Ti microscope (Nikon, Tokyo, Japan). Nuclei were visualized by mounting the sections in 4′,6-diamidino-2-phenylindole (DAPI)-containing VECTASHIELD Mounting Medium (VectorLabs, Newark, CA). Labeled tissues were visualized using the Nikon Eclipse Ti confocal microscope.

### Statistical Analysis

All data are expressed as mean ± SEM unless indicated otherwise. Where more than two treatment conditions were compared, one-way or two-way analysis of variance (ANOVA) was used, followed by a post hoc comparison test, as indicated. Where only two treatment conditions were compared, the significance of differences between the two conditions was determined by the two-tailed Student’s *t*-test. The 50% lethal dose (LD_50_) was determined by nonlinear fit regression analysis (variable slope). All statistical procedures were done using Prism 9 (GraphPad, Boston, MA). Statistical significance was defined as *P* < 0.05.

## Results

### Evaluation of Dose and VPA and MMC Treatment on Cultured Primary HTF Viability

To assess if utilizing a combination of VPA and MMC on primary HTFs induced unwanted synergistic cytotoxic effects, we performed dose escalation assays to assess the toxic effect of VPA and MMC treatment individually, as well as their combination (Fig. 2). Due to the highly cytotoxic nature of MMC, cells were exposed to it transiently and it was then washed from the media, as described above. The transient exposure to MMC mirrors the transient MMC exposure by sponge application in glaucoma filtration surgery. The growth rate of HTFs was measured by xCELLigence real-time cell analysis. As expected, brief exposure to MMC led to dose-dependent inhibition of cell growth (Fig. 2A). In contrast, VPA led to a reduction in cell growth at a much higher dose (3 mg/mL) (Fig. 2B). The combination of VPA and MMC demonstrated no significant changes in cell growth (Figs. 2C–2F), indicating that the combined treatment does not cause more cytotoxic effects beyond what was anticipated from each treatment alone.

These results confirmed that VPA has a significantly better safety profile compared to MMC. In addition, the combination of VPA and MMC did not result in a synergistic increase of toxicity beyond what is expected from separate applications of MMC and VPA. Taken together, the combination of VPA and low-dose MMC provided the optimal condition of reduced collagen-induced scarring on human fibroblasts with minimal toxicity.

### Effect of MMC and VPA Combination on Collagen Expression in the Rabbit MIBS Model

We have previously shown that the combination of VPA and MMC resulted in a lower *Col1a1* expression in the bleb tissues of the rabbit MIBS model compared to MMC treatment alone.^11^ In this study, to determine the optimal route and frequency of administration for future clinical use, we compared the efficacy of different administration modes of follow-up VPA treatment. Follow-up VPA treatment was administered in two ways: weekly SC injection (0.1 mL of 1 mg/mL) at days 7, 14, and 21 or topical instillation of VPA eye drops (5% w/v) four times daily from day 1 to day 28.

**Figure 3.**
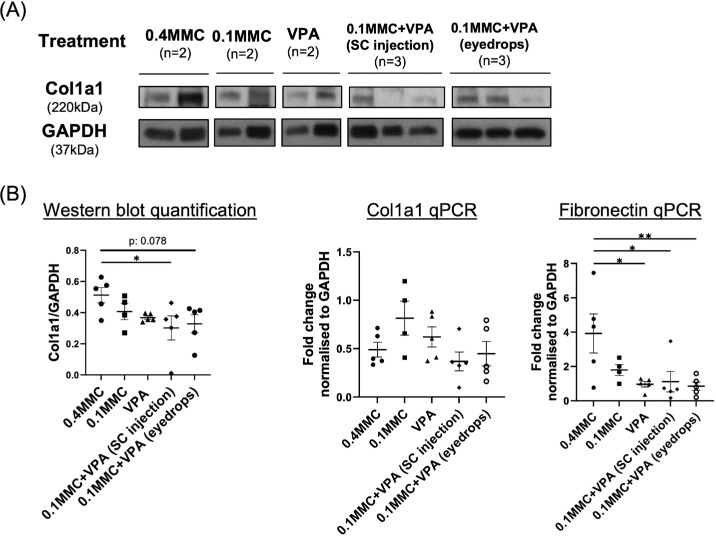
Combination of low-dose MMC and VPA reduced ECM expression in bleb tissues of the rabbit MIGS model. Rabbit conjunctiva tissues from both contralateral unoperated and implanted eyes were harvested on day 28 after surgery and were subjected to analyses by immunoblot for type I collagen and real-time PCR for *Col1a1* and *Fn* genes. Treatment conditions are as described in the Materials and Methods section (rabbit model of MIBS) and in Figure 1 (Study 1). Briefly, 0.4MMC refers to the treatment with 0.4-mg/mL MMC only (*n* = 5); 0.1MMC refers to the treatment with 0.1mg/mL MMC only (*n* = 4); VPA refers to the treatment with SC injection of 0.1 mL of 1-mg/mL VPA at the following postoperative time points: days 0, 1, 2, 3, 4, 5, 6, 7, 10, 14, and 21 (*n* = 5); 0.1MMC+VPA (SC injection) refers to the combination treatment of SC VPA 1 mg/mL with 0.1-mg/mL MMC with follow-up weekly injections of 0.1-mg/mL VPA (*n* = 5); 0.1MMC+VPA (eyedrops) refers to the combination of SC VPA 2 mg/mL with 0.1-mg/mL MMC with follow-up daily 5% VPA eye drops four times a day (*n* = 5). (**A**) Representative *Col1a1* and *Gapdh* immunoblots from implanted eyes from one experiment are shown in the *top panel*. Representative sample duplicates or triplicates from each experiment group are shown as indicated. The *bottom left panel* shows densitometry values of *Col1a1*, normalized to GAPDH. (**B**) Real-time PCR analysis of the values shown were calculated as fold changes from contralateral unoperated levels. The graph represents the mean ± SD. Each symbol represents one rabbit eye. *P was determined by one-way ANOVA followed by Dunnett's multiple-comparison test.

We assessed the expression level of extracellular matrix molecules (*Col1a1* and fibronectin) in the bleb tissues 28 days after surgery. Consistent with our previous study, the combination of VPA and MMC significantly reduced *Col1a1* protein levels compared to the MMC monotherapy group. Importantly, postoperative VPA administration by weekly injection or by topical instillation was shown to be equally effective in reducing collagen expression levels in the bleb tissues (Fig. 3A).

In addition to the collagen protein level, the mRNA expression level of *Col1a1* and fibronectin genes was also assessed (Fig. 3B). Postoperative treatment with both VPA eye drops and SC injection of VPA resulted in a reduction in *Col1a1* mRNA and protein expression, although statistical significance was only seen in the SC injection group. The changes in fibronectin mRNA expression appeared consistent with the reduced collagen protein level, indicating that the combination of VPA and MMC in both modes of administration, topical and injection, resulted in an equally effective reduction of extracellular matrix (ECM) gene expression in the blebs in this model. Consistent with in vitro observations, the TUNEL assay (red) revealed no degraded DNA, and DAPI staining (blue) showed no changes in nuclear morphology in the conjunctiva across all treatment groups (Supplementary Fig. S1).

### Effect of Administration of MMC and VPA Combination on Postoperative Collagen Organization

**Figure 4.**
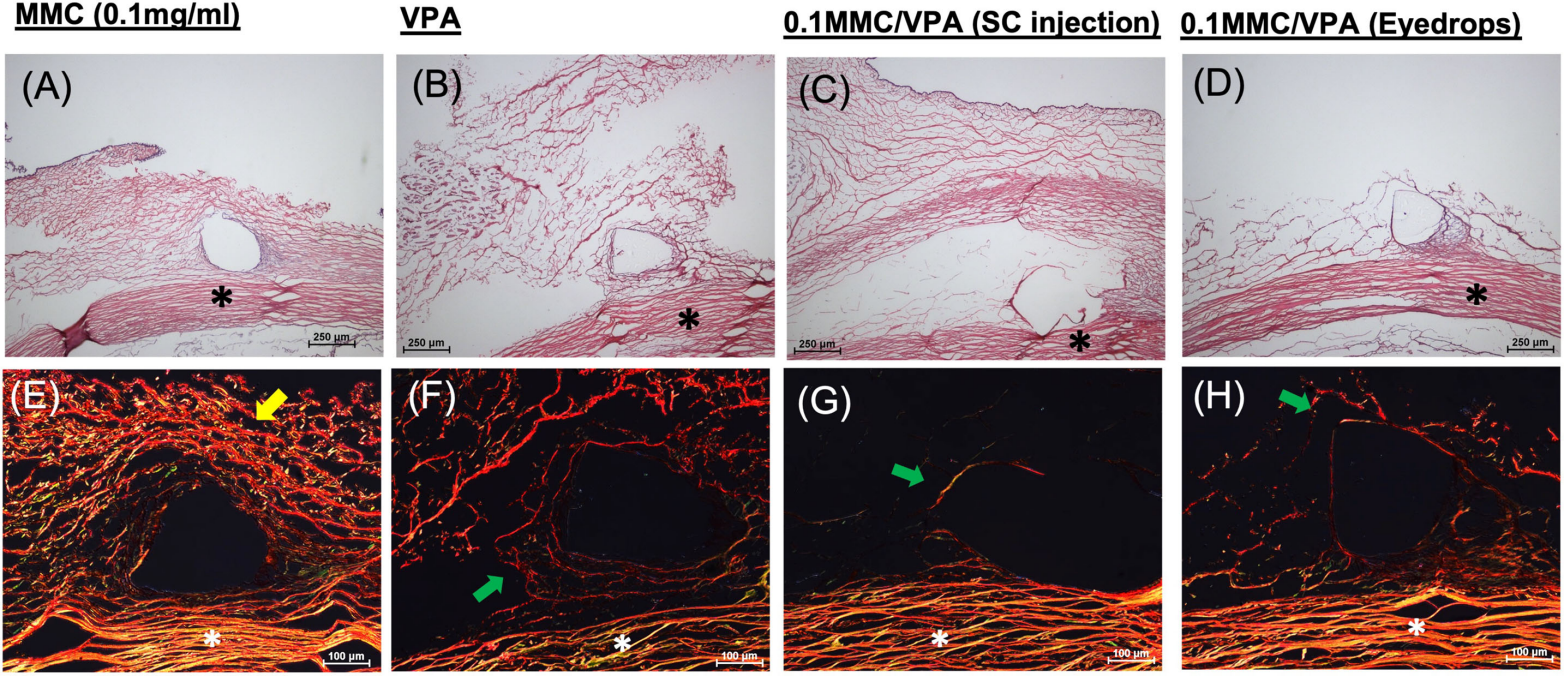
Histochemical visualization of collagen characteristics in the rabbit MIGS model treated with a combination of low-dose MMC and VPA. Serial frozen sections of the day 28 postoperative conjunctiva from a representative eye from each experimental group were visualized via the indicated H&E (*top*) and Picosirius red (*bottom*) staining. The cryosections stained with H&E were viewed under light microscopy, and the cryosections stained with Picrosirius red were viewed via polarized microscopy. The *yellow arrow* indicates mature thick collagen fibers, and the *green arrow* indicates immature thin collagen fibrils. *Black asterisks* (*) indicate the sclera. *Scale bar*: 100 µm. Treatment conditions are as described in the Materials and Methods section (rabbit model of MIBS) and in Figure 1 (Study 1). (**A**, **E**) Treatment group with 0.1-mg/mL MMC only (*n* = 4). (**B**, **F**) Treatment group with SC injection of 0.1 mL of 1-mg/mL VPA at the following postoperative time points: days 0, 1, 2, 3, 4, 5, 6, 7, 10, 14, and 21 (*n* = 5). (C, G) Combination treatment group of subconjunctival VPA 1 mg/mL with 0.1-mg/mL MMC with follow-up weekly injection of 0.1-mg/mL VPA (*n* = 5). (**D**, **H**) Combination treatment group of subconjunctival VPA 1 mg/mL with 0.1-mg/mL MMC with follow-up daily 5% VPA eye drops four times a day (*n* = 5).

We have previously shown that, in blebs treated with high-dose MMC (0.4 mg/mL), ECM structure is largely obliterated, resulting in large voids.^11^ The polarized colors of Picosirius red–stained fibrillar collagen are dependent on fiber thickness and packing and may be used to differentiate thin, newly formed green/yellow collagen fibers from thick, mature, orange/red collagen bundles.^14^ Although ECM in the blebs treated with low-dose MMC (0.1 mg/mL) (Fig. 4A) retained a fibrous network of mature thick collagen fibers that permeated throughout the stromal matrix, the blebs treated with VPA alone, as well as the MMC+VPA combination, in contrast, contained predominantly immature thin collagen fibrils (Figs. 4B–4D). These fibers were much finer compared with those receiving MMC monotherapy. These data suggest that incorporation of the VPA either by SC injection or administered topically, could effectively prevent excessive mature collagen fiber formation, maintaining a favorable ECM organization within the bleb.

### Comparison of the Effect of VPA Administration by SC Injection and Topical Routes on IOP Lowering

**Figure 5.**
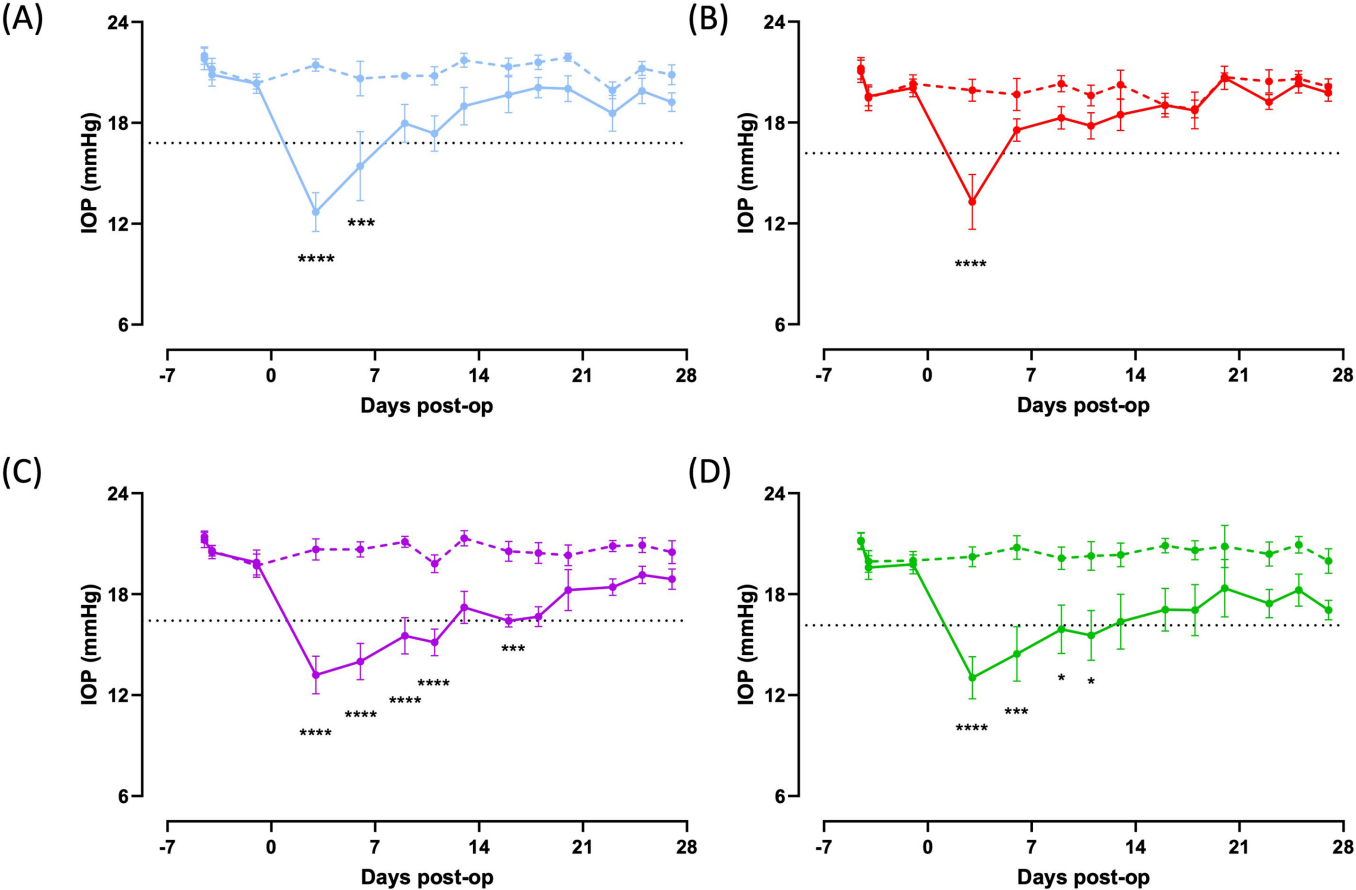
IOP of rabbit MIGS model treated with low-dose MMC and VPA combination. IOP was measured with a pneumatonometer on conscious rabbits twice weekly up to 28 days after MIGS surgery. Mean IOP ± SEM of the operated (*solid line*) and unoperated contralateral (*dashed* *line*) lines are shown for each experimental group, as described in the Materials and Methods section. The *dotted line* indicates the level where IOP is 20% lower than the baseline. *P was determined by two-way ANOVA followed by Sidak's multiple-comparison test. Treatment conditions are as described in the Materials and Methods section (rabbit model of MIBS) and in Figure 1 (Study 2). (**A**) Treatment group with 0.1-mg/mL MMC only (*n* = 5). (**B**) Treatment group with SC injection of 0.1 mL of 30-mg/mL VPA given intraoperatively without further treatment (*n* = 6). (**C**) Combination treatment group of subconjunctival VPA 30 mg/mL with 0.1-mg/mL MMC with follow-up weekly injections of 10-mg/mL VPA (*n* = 6). (**D**) Combination treatment group of subconjunctival VPA 30 mg/mL with 0.1-mg/mL MMC with follow-up daily 15% VPA eye drops six times a day (*n* = 6).

To optimize the anti-scarring effect of VPA to maintain optimal bleb function, a second study with a higher VPA concentration was performed. We determined the highest dose of VPA administered either topically or by SC injection based on the solubility of the formulation as well as the tolerability of the drug on the ocular surface. Topical drops were given six times a day to maximize the frequency of drops (assuming a high wash-over period at the tear-film interface) while balancing the frequency of drops a patient can feasibly instill over 24 hours. To assess the functionality of draining blebs, IOP was measured on the operated eye as well as the untreated contralateral eye twice weekly after surgery for up to 4 weeks (Fig. 5, Supplementary Table S1). There was no statistical significance in the preoperative IOP among all of the experimental groups. Eyes that were treated with a single injection of VPA showed a transient IOP lowering on day 3, followed by a small degree of IOP reduction up to day 14, which was not statistically significant. Eyes that were treated with 0.1-mg/mL MMC (Fig. 5A) demonstrated a more sustained IOP lowering, which was statistically significant compared to the contralateral eye up to day 9. Although the injection of VPA alone did not show a sustained IOP lowering effect, the addition of VPA treatment through either SC injection (Fig. 5C) or topical instillation (Fig. 5D) induced a stronger and more sustainable IOP lowering. This was statistically significant up to day 20 and day 13, respectively. The result confirms that, in this model, the addition of VPA with low-dose MMC, either by SC injection or by topical instillation, allows for more effective and sustained IOP management.

## Discussion

Our study explored possible administration routes of VPA in combination with low-dose MMC. Weekly subconjunctival injections of VPA and daily VPA eye drops have both shown reduced expression of both collagen I and fibronectin levels. When used in combination with MMC, VPA was effective in maintaining postoperative bleb vasculature and reducing encapsulation while maintaining an excellent safety profile. In addition, this combination also demonstrated a sustained IOP-lowering effect.

VPA has been used in neuropsychiatric disorders over the past 40 years, and its safety profile has been well documented.^15^ A repurposed new indication as an antifibrotic has since been discovered, as it has shown the capacity to downregulate selective proinflammatory cytokines involved in inhibiting inflammation and fibrosis.^16^ Systemically, VPA has been shown to inhibit fibrosis in the liver, kidney, and heart through different mechanisms of attenuating transforming growth factor beta (TGF-β) expression and reducing macrophage infiltration.^17^ In ophthalmology, previous studies have demonstrated that VPA is an effective inhibitor of type I collagen seen in conjunctival fibrosis.^13^ This finding was later reiterated in a rabbit MIBS model, showing that VPA modulated collagen deposition, modifying scar structure to favor filtration in glaucoma patients.^11^

Histological visualization of blebs treated with VPA and low-dose MMC has shown thin immature collagen fibrils. This finding was maintained across the two different routes of administration. Collagen 1a1 is overexpressed in the early and fibrotic phases of wound healing in glaucoma filtration surgery (GFS).^18^ Significantly high levels of *Col1a1* mRNAs were found in conjunctival tissues of patients who had failed glaucoma surgeries compared to patients who had not previously undergone surgery.^18^ VPA can exert posttranslational modification of secreted *Col1a1* that results in finer immature fibers in the blebs after surgery. This further supports our notion that VPA, in part through this approach, can help to maintain a favorable bleb profile and function.

Clinically, prolonging bleb function in a failing bleb via bleb needling with adjunctive anti-metabolite agents such as MMC or 5-fluorouracil (5-FU) has vastly improved the success rates of glaucoma filtration surgery. However multiple needling procedures are often required to achieve long-term bleb function stability. A 3-year study on a single bleb needling procedure with adjunctive MMC reported a success rate of 36%, with half of the cohort requiring further surgery or laser.^19^ Maestrini et al.^20^ reported better bleb survival rates when multiple needling procedures were performed. MMC or 5-FU, however, has been associated with corneal erosions, bleb leaks, and cystic, avascular, thin-walled blebs.^21^^,^^22^ Repeated doses of these adjuncts in bleb needling procedures can lead to more serious sight-threatening complications such as blebitis and endophthalmitis.^5^

Uniquely, our study explored the potential diverse clinical utility of having different applications of VPA in the context of bleb-forming surgeries that also include MicroShunt implantation. With subconjunctival injections, a higher drug distribution in the anterior chamber is attained. Drug bioavailability in the aqueous humor is reported to be 10% after subconjunctival injection, as it bypasses the corneal barrier and is retained longer in the injection site after administration.^23^ In contrast, topical applications have the advantages of being less invasive and a more familiar route for patients.

It has been reported that one of the significant risk factors contributing to surgical failure is the use of preoperative anti-glaucoma topical therapy.^24^ Bonomi et al.^25^ conducted a study that showed the risk of surgical failure doubled after 8 years of cumulative topical therapy. Monocyte chemoattractant protein-1 (MCP-1), a marker mediating leukocyte recruitment to sites of inflammation in the early stages of wound healing, is high in chronically inflamed eyes.^26^ In glaucoma, MCP-1 was found to be significantly elevated in medicated versus unmedicated eyes, as well as in eyes with a higher risk of surgical failure.^26^ Anti-glaucoma drops cause a change in the conjunctiva that seems to have a deleterious effect on the outcome of glaucoma surgery.^24^ Broadway et al.^27^ ran a study to see if these effects could be reversed. Anti-glaucoma drops were stopped, and a corticosteroid was started 1 month preoperatively. It was found that the number of fibroblasts and inflammatory cells in the conjunctiva decreased and, inversely, the success rates of filtration surgery increased.^27^ VPA has been shown to exhibit characteristics of inhibiting several proinflammatory cytokines in the operated conjunctiva.^16^ Therefore, VPA as a topical formulation, may have a role as an eyedrop as a preoperative regimen to improve the success rate of glaucoma surgery.

A combination of drug delivery approaches can therefore help to achieve optimal anti-inflammatory and antifibrotic effects, thereby prolonging surgical success. Pakravan et al.^28^ conducted a study comparing the safety and efficacy of subconjunctival 5-FU versus topical MMC for the management of early bleb failure after GFS and reported comparable outcomes. Clinically, VPA can be administered subconjunctivally intraoperatively and postoperatively, in addition to topical administration. VPA therefore allows flexibility in the mode of its delivery to address different phases of conjunctival wound healing in the context of an eye undergoing filtration surgery. In addition, both subconjunctival and topical formulations have demonstrated an excellent safety profile with no evidence of toxicity along with good tolerability in the animals. We demonstrated that, at maximum tolerability and solubility, VPA administration by either weekly subconjunctival injection or daily eye drop instillation resulted in similar effectiveness in maintaining favorable bleb characteristics associated with IOP reduction. Despite the two differing routes of administration, there were no overt side effects seen throughout our experiments at these doses or dosing regimens.

With an expanded surgical repertoire in glaucoma, we see an increased need for a new antifibrotic that is not only efficacious but also safe. We see MIBS being increasingly used, as it has a better safety profile; however, a higher dose of MMC is required to prevent failure.^29^ Our study evaluated different routes of VPA administration in combination with low-dose MMC following experimental implantation of the PRESERFLO MicroShunt in rabbits. There are, however, limitations with regard to the short study duration of 28 days. Further studies are required to optimize the topical and injection frequency of VPA, as well as for formulation refinement. The clinical utility of pretreatment with VPA should also be explored, as well as rescuing a failing bleb, to understand the full potential benefits of VPA in the postoperative management of bleb filtration surgeries.

In conclusion, our study provides insights into the versatility of VPA as an antifibrotic agent that allows for different modalities of application to target ECM remodeling during the late stages of filtration surgery wound healing. Application of lower concentrations of MMC or entirely removing its use may be a possibility with adjunctive VPA.

## Supplementary Material

Supplement 1

## References

[1] Chan PPM, Larson MD, Dickerson JE Jr, et al. Minimally invasive glaucoma surgery: latest developments and future challenges. *Asia-Pac J Ophthalmol Phila Pa*. 2023; 12: 537–564. 10.1097/APO.0000000000000646 38079242

[2] Pinchuk L, Riss I, Batlle JF, et al. The development of a micro-shunt made from poly(styrene-block-isobutylene-block-styrene) to treat glaucoma. *J Biomed Mater Res B Appl Biomater*. 2017; 105: 211–221. 26380916 10.1002/jbm.b.33525 PMC5215625

[3] Mearza AA, Aslanides IM. Uses and complications of mitomycin C in ophthalmology. *Expert Opin Drug Saf*. 2007; 6: 27–32. 17181449 10.1517/14740338.6.1.27

[4] Seet L-F, Lee WS, Su R, Finger SN, Crowston JG, Wong TT. Validation of the glaucoma filtration surgical mouse model for antifibrotic drug evaluation. *Mol Med Camb Mass*. 2011; 17: 557–567. 21229189 10.2119/molmed.2010.00188 PMC3105140

[5] Anand N, Arora S, Clowes M. Mitomycin C augmented glaucoma surgery: evolution of filtering bleb avascularity, transconjunctival oozing, and leaks. *Br J Ophthalmol*. 2006; 90: 175–180. 16424529 10.1136/bjo.2005.077800 PMC1860189

[6] Occleston NL, Daniels JT, Tarnuzzer RW, et al. Single exposures to antiproliferatives: long-term effects on ocular fibroblast wound-healing behavior. *Invest Ophthalmol Vis Sci*. 1997; 38: 1998–2007. 9331263

[7] Holló G . Wound healing and glaucoma surgery: modulating the scarring process with conventional antimetabolites and new molecules. *Dev Ophthalmol*. 2017; 59: 80–89. 28442689 10.1159/000458488

[8] Dave B, Patel M, Suresh S, et al. Wound modulations in glaucoma surgery: a systematic review. *Bioengineering*. 2024; 11: 446. 38790314 10.3390/bioengineering11050446 PMC11117829

[9] Khan S, Ahirwar K, Jena G. Anti-fibrotic effects of valproic acid: role of HDAC inhibition and associated mechanisms. *Epigenomics*. 2016; 8: 1087–1101. 27411759 10.2217/epi-2016-0034

[10] Yap ZL, Seet L-F, Chu SWL, Toh LZ, Ibrahim FI, Wong TT. Effect of valproic acid on functional bleb morphology in a rabbit model of minimally invasive surgery. *Br J Ophthalmol*. 2022; 106: 1028–1036. 34266858 10.1136/bjophthalmol-2020-318691 PMC9234410

[11] Seet L-F, Yap ZL, Chu SWL, et al. Effects of valproic acid and mitomycin C combination therapy in a rabbit model of minimally invasive glaucoma surgery. *Transl Vis Sci Technol*. 2022; 11: 30. 10.1167/tvst.11.1.30 PMC8787605 35044442

[12] Khaw PT, Ward S, Porter A, Grierson I, Hitchings RA, Rice NS. The long-term effects of 5-fluorouracil and sodium butyrate on human Tenon's fibroblasts. *Invest Ophthalmol Vis Sci*. 1992; 33: 2043–2052. 1582809

[13] Seet L-F, Toh LZ, Finger SN, Chu SWL, Stefanovic B, Wong TT. Valproic acid suppresses collagen by selective regulation of Smads in conjunctival fibrosis. *J Mol Med Berl Ger*. 2015; 94: 321–324. 10.1007/s00109-015-1358-z PMC4803820 26507880

[14] Piérard GE . Sirius red polarization method is useful to visualize the organization of connective tissues but not the molecular composition of their fibrous polymers. *Matrix Stuttg Ger*. 1989; 9: 68–71. 10.1016/s0934-8832(89)80021-6 2710035

[15] Rosenberg G . The mechanisms of action of valproate in neuropsychiatric disorders: can we see the forest for the trees? *Cell Mol Life Sci*. 2007; 64: 2090–2103. 17514356 10.1007/s00018-007-7079-x PMC11149473

[16] Seet L-F, Toh LZ, Finger SN, Chu SWL, Wong TT. Valproic acid exerts specific cellular and molecular anti-inflammatory effects in post-operative conjunctiva. *J Mol Med Berl Ger*. 2019; 97: 63–75. 10.1007/s00109-018-1722-x PMC6326969 30456449

[17] Costalonga EC, de Freitas LJ, da Aragone DSP, Silva FMO, Noronha IL. Anti-fibrotic effects of valproic acid in experimental peritoneal fibrosis. *PLoS One*. 2017; 12: e0184302. 28873458 10.1371/journal.pone.0184302 PMC5584960

[18] Seet L-F, Toh LZ, Chu SWL, Finger SN, Chua JLL, Wong TT. Upregulation of distinct collagen transcripts in post-surgery scar tissue: a study of conjunctival fibrosis. *Dis Model Mech*. 2017; 10: 751–760. 28331057 10.1242/dmm.028555 PMC5483006

[19] Lin S, Byles D, Smith M. Long-term outcome of mitomycin C-augmented needle revision of trabeculectomy blebs for late trabeculectomy failure. *Eye (Lond)*. 2018; 32: 1893–1899. 30158576 10.1038/s41433-018-0199-8 PMC6292892

[20] Maestrini HA, Cronemberger S, Matoso HDS, et al. Late needling of flat filtering blebs with adjunctive mitomycin C: efficacy and safety for the corneal endothelium. *Ophthalmology*. 2011; 118: 755–762. 21055818 10.1016/j.ophtha.2010.08.020

[21] Franks WA, Hitchings RA. Complications of 5-fluorouracil after trabeculectomy. *Eye (Lond)*. 1991; 5: 385–389. 1743353 10.1038/eye.1991.63

[22] Lama PJ, Fechtner RD. Antifibrotics and wound healing in glaucoma surgery. *Surv Ophthalmol*. 2003; 48: 314–346. 12745005 10.1016/s0039-6257(03)00038-9

[23] ScienceDirect. Subconjunctival drug administration. Available at: https://www.sciencedirect.com/topics/medicine-and-dentistry/subconjunctival-drug-administration. Accessed May 14, 2025.

[24] Broadway D, Grierson I, Hitchings R. Adverse effects of topical antiglaucomatous medications on the conjunctiva. *Br J Ophthalmol*. 1993; 77: 590–596. 8218059 10.1136/bjo.77.9.590 PMC513958

[25] Bonomi L, Zavarise G, Noya E, Michieletto S. Effects of timolol maleate on tear flow in human eyes. *Albrecht Von Graefes Arch Klin Exp Ophthalmol*. 1980; 213: 19–22. 6906142 10.1007/BF02391207

[26] Chong RS, Jiang YZ, Boey PY, et al. Tear cytokine profile in medicated glaucoma patients: effect of monocyte chemoattractant protein 1 on early posttrabeculectomy outcome. *Ophthalmology*. 2010; 117: 2353–2358. 20630596 10.1016/j.ophtha.2010.03.064

[27] Broadway DC, Grierson I, Stürmer J, Hitchings RA. Reversal of topical antiglaucoma medication effects on the conjunctiva. *Arch Ophthalmol*. 1996; 114: 262–267. 8600884 10.1001/archopht.1996.01100130258004

[28] Pakravan M, Miraftabi A, Yazdani S, Koohestani N, Yaseri M. Topical mitomycin-C versus subconjunctival 5-fluorouracil for management of bleb failure. *J Ophthalmic Vis Res*. 2011; 6: 78–86. 22454715 PMC3306090

[29] Bell K, de Padua Soares Bezerra B, Mofokeng M, et al. Learning from the past: mitomycin C use in trabeculectomy and its application in bleb-forming minimally invasive glaucoma surgery. *Surv Ophthalmol*. 2021; 66: 109–123. 32450159 10.1016/j.survophthal.2020.05.005

