# Supplementary material for: Valproic Acid Application to Modify Post Surgical Fibrosis in a Model of Minimally Invasive Bleb Surgery
Source: Transl Vis Sci Technol. 2025 Jun 2;14(6):6. doi: 10.1167/tvst.14.6.6 (PMC12136099; doi:10.1167/tvst.14.6.6)
Supplement: Supplement 1 [file tvst-14-6-6_s001.pdf]

## Supplemental Figure 1:

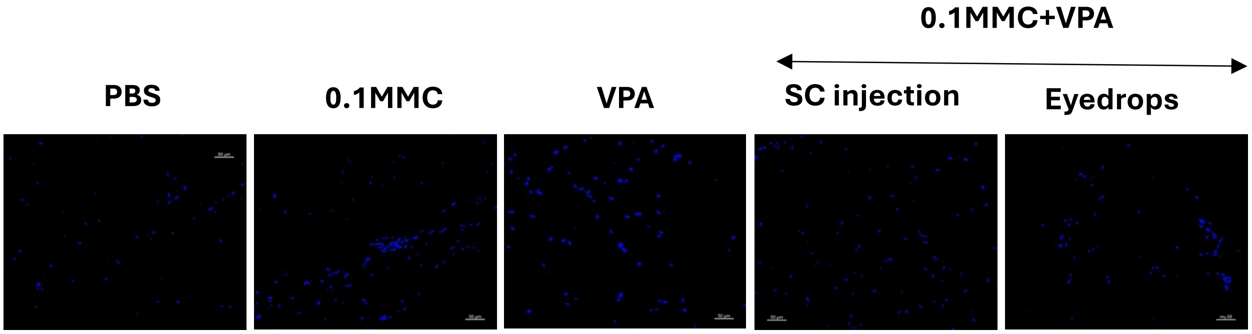


*Figure 1: TUNEL assay of rabbit MIGS model treated with low dose MMC and VPA combination.* Representative images of rabbit eye cross-sections labeled with TUNEL assay (red) and corresponding DAPI staining (blue) at the experiment endpoint, demonstrating no change in the morphology of nuclei and no presence of the degraded DNA.

## Supplemental Table 1: IOP values

| Days post-surgery | PBS | | | | 0.1 MMC | | | | VPA | | | | 0.1MMC+VPA (SC injection) | | | | 0.1MMC+VPA (Topical) | | | |
|---|---|---|---|---|---|---|---|---|---|---|---|---|---|---|---|---|---|---|---|---|
| | Con eye | | Op eye | | Con eye | | Op eye | | Con eye | | Op eye | | Con eye | | Op eye | | Con eye | | Op eye | |
| | mean | SD | mean | SD | mean | SD | mean | SD | mean | SD | mean | SD | mean | SD | mean | SD | mean | SD | mean | SD |
| -4.5 | 21.36 | 1.64 | 20.95 | 1.18 | 22.00 | 1.16 | 21.80 | 1.44 | 21.22 | 1.58 | 21.06 | 1.63 | 21.42 | 0.84 | 21.22 | 1.11 | 21.14 | 1.21 | 21.19 | 1.18 |
| -4 | 20.89 | 1.45 | 19.78 | 2.36 | 21.20 | 1.43 | 20.87 | 1.54 | 19.47 | 1.89 | 19.56 | 1.40 | 20.58 | 0.77 | 20.50 | 0.95 | 19.95 | 1.58 | 19.59 | 1.72 |
| -1 | 19.81 | 1.71 | 19.64 | 1.63 | 20.37 | 0.80 | 20.34 | 1.28 | 20.31 | 1.31 | 20.06 | 1.29 | 19.70 | 1.69 | 19.89 | 1.81 | 20.00 | 1.31 | 19.78 | 1.40 |
| 3 | 20.06 | 2.25 | 14.26 | 1.78 | 21.43 | 0.84 | 12.70 | 2.59 | 19.92 | 1.60 | 13.28 | 4.00 | 20.67 | 1.55 | 13.19 | 2.74 | 20.22 | 1.44 | 13.03 | 3.07 |
| 6 | 20.50 | 1.92 | 17.64 | 2.02 | 20.63 | 2.32 | 15.43 | 4.60 | 19.67 | 2.34 | 17.55 | 1.64 | 20.67 | 1.12 | 14.00 | 2.64 | 20.78 | 1.72 | 14.44 | 3.98 |
| 9 | 19.83 | 1.85 | 19.31 | 1.98 | 20.80 | 0.40 | 17.97 | 2.51 | 20.31 | 1.17 | 18.28 | 1.65 | 21.11 | 0.80 | 15.53 | 2.65 | 20.14 | 1.64 | 15.92 | 3.55 |
| 11 | 19.17 | 1.75 | 18.67 | 2.38 | 20.80 | 1.19 | 17.37 | 2.34 | 19.61 | 1.50 | 17.81 | 1.90 | 19.81 | 1.29 | 15.14 | 1.94 | 20.28 | 2.08 | 15.56 | 3.64 |
| 13 | 20.56 | 1.57 | 20.03 | 1.39 | 21.73 | 0.94 | 19.00 | 2.51 | 20.25 | 2.13 | 18.47 | 2.29 | 21.33 | 1.12 | 17.22 | 2.34 | 20.33 | 1.73 | 16.36 | 4.00 |
| 16 | 20.47 | 1.34 | 20.03 | 1.56 | 21.33 | 1.16 | 19.67 | 2.39 | 19.03 | 1.20 | 19.03 | 1.72 | 20.56 | 1.45 | 16.42 | 0.89 | 20.89 | 1.05 | 17.09 | 3.10 |
| 18 | 19.42 | 2.32 | 19.08 | 2.44 | 21.60 | 0.96 | 20.10 | 1.33 | 18.81 | 1.29 | 18.72 | 2.68 | 20.45 | 1.52 | 16.67 | 1.45 | 20.61 | 1.40 | 17.06 | 3.72 |
| 20 | 21.09 | 1.31 | 20.81 | 1.91 | 21.90 | 0.54 | 20.03 | 1.71 | 20.70 | 0.61 | 20.67 | 1.69 | 20.31 | 1.53 | 18.25 | 2.98 | 20.83 | 3.08 | 18.36 | 4.17 |
| 23 | 19.39 | 1.34 | 19.28 | 1.16 | 19.93 | 1.12 | 18.57 | 2.37 | 20.44 | 1.70 | 19.22 | 1.08 | 20.86 | 0.79 | 18.42 | 1.21 | 20.39 | 1.74 | 17.45 | 2.05 |
| 25 | 20.45 | 0.81 | 19.89 | 0.91 | 21.23 | 0.93 | 19.90 | 1.69 | 20.61 | 1.14 | 20.31 | 1.36 | 20.92 | 1.09 | 19.14 | 1.26 | 20.94 | 1.21 | 18.25 | 2.35 |
| 27 | 19.17 | 1.25 | 19.00 | 2.22 | 20.87 | 1.33 | 19.23 | 1.22 | 20.14 | 1.13 | 19.78 | 1.24 | 20.50 | 1.66 | 18.89 | 1.50 | 19.97 | 1.77 | 17.06 | 1.43 |

Con eye: untreated contralateral eye
Op eye: operated eye
Units are in mmHg
